# Public health emergency decision-making and management system sound research using rough set attribute reduction and blockchain

**DOI:** 10.1038/s41598-022-07493-w

**Published:** 2022-03-04

**Authors:** Hanyi Wang

**Affiliations:** School of Economics and Management, Kunming University, Kunming, 650214 China

**Keywords:** Musculoskeletal system, Predictive markers

## Abstract

Public health emergency decisions are explored to ensure the emergency response measures in an environment where various emergencies occur frequently. An emergency decision is essentially a multi-criteria risk decision-making problem. The feasibility of applying prospect theory to emergency decisions is analyzed, and how psychological behaviors of decision-makers impact decision-making results are quantified. On this basis, the cognitive process of public health emergencies is investigated based on the rough set theory. A Decision Rule Extraction Algorithm (denoted as A-DRE) that considers attribute costs is proposed, which is then applied for attribute reduction and rule extraction on emergency datasets. In this way, decision-makers can obtain reduced decision table attributes quickly. Considering that emergency decisions require the participation of multiple departments, a framework is constructed to solve multi-department emergency decisions. The technical characteristics of the blockchain are in line with the requirements of decentralization and multi-party participation in emergency management. The core framework of the public health emergency management system-plan, legal system, mechanism, and system can play an important role. When $\delta { = }0.10$, the classification accuracy under the K-Nearest Neighbor (KNN) classifier reaches 73.5%. When $\delta { = }0.15$, the classification accuracy under the Support Vector Machines (SVM) classifier reaches 86.4%. It can effectively improve China’s public health emergency management system and improve the efficiency of emergency management. By taking Coronavirus Disease 2019 (COVID-19) as an example, the weight and prospect value functions of different decision-maker attributes are constructed based on prospect theory. The optimal rescue plan is finally determined. A-DRE can consider the cost of each attribute in the decision table and the ability to classify it correctly; moreover, it can reduce the attributes and extract the rules on the COVID-19 dataset, suitable for decision-makers' situation face once an emergency occurs. The emergency decision approach based on rough set attribute reduction and prospect theory can acquire practical decision-making rules while considering the different risk preferences of decision-makers facing different decision-making results, which is significant for the rapid development of public health emergency assistance and disaster relief.

## Introduction

Coronavirus Disease 2019 (COVID-19) is a major public health emergency with the fastest spread, the widest range of infections, and the most difficult prevention and control since founding the People’s Republic of China^1^. It poses a major challenge to China’s medical and healthcare system and significantly affects China’s economy and society. The National Natural Science Foundation of China has launched a special project to provide decision-making support and countermeasures for major public health emergencies, such as scientific prevention and control and response to COVID-19, to reduce the impact of diseases on the national economy and society. At present, COVID-19 continues to spread globally, posing a major challenge to the global medical and healthcare system while promoting the reform of the global governance system^2–4^. The World Health Organization (WHO) has held its seventh conference of the COVID-19 Emergency Committee on April 15, 2021, to assess the global situation, provide corresponding suggestions, and discuss the vaccines and new variants of COVID-19 investigate the healthcare measures of international travels^5^. The committee also recommends strengthening epidemiological and virological surveillance, encouraging genetic sequencing of COVID-19, and sharing the research data.

Based on the spread of COVID-19 this time, establishing a sound local and national response mechanism for public health emergencies is the key to controlling the further spread of public health emergencies and reducing their losses. Early detection, early reporting, early investigation, and early response are the basic principles of handling public health emergencies^6,7^. Any behavior that blocks public health emergency reporting channels and intercepts information about emergencies will fundamentally shake the public health emergency response mechanism. The attributes of public health emergency have the characteristics of dynamic derivation and uncertainty, such as natural disasters, infectious diseases, mass diseases, and major food poisoning. Hence, while formulating an emergency decision scheme, combining events such as multi-attribute, multi-scenario, and multi-stage factors is necessary to construct the corresponding decision-making scheme in each stage. The multi-stage and the multi-department emergency decision can be abstractly constructed into a collaborative emergency decision model to integrate the opinions of different departments to deal with dynamic events. Psychological behavior plays a vital role during decision-making. As a psychological theory, prospect theory believes that everyone will have different attitudes towards risk based on different initial conditions^8,9^. Zhang et al.^10^ considered the psychological behavior of decision-makers and applied prospect theory to emergency treatment. This theory could produce better solutions for different emergencies. Blockchain can fully assess risks and improve emergency plans to improve the national public health emergency management system. It can improve the comprehensiveness and scientific nature of legislative evaluation, jointly build alliance chains with law enforcement agencies to realize automatic supervision on the chain, and keep evidence of behavior on the chain, leaving traces in the law enforcement process. Blockchain can further improve the emergency management mechanism to ensure that there are facts for monitoring in advance, measures during the event have a basis, and the system after the event is improved. This provides a scientific basis for deepening system reform. Khurshid^11^ described how blockchain offers a solution to data-related trust problems with its distributed trust network and cryptography-based security. Blockchain can positively change the nature of trust, value sharing, and transactions by relying on a distributed, robust, secure, privacy-preserving, and immutable record-keeping framework.

In the existing research, the formulation of emergency decision-making for public health emergencies needs to combine many factors of multi-attribute, multi-scenario and multi-stage. But there is a lack of discussion about the security of decisions made by various departments. The technical characteristics of the blockchain are considered to meet the requirements of decentralized and multi-party participation in emergency management. Therefore, blockchain technology can play an essential role in the core framework plan, legal system, mechanism, and public health emergency management system. Moreover, factors that cause emergencies are very complex. Due to huge amounts of data, diverse information types, and lack of information, the cost should be considered when acquiring corresponding knowledge to effectively deal with various ambiguities and missing information. Hence, attribute reduction and rule extraction are first applied to explore an event's knowledge recognition process based on the rough set theory. Second, a multi-attribute emergency decision model is constructed based on prospect theory to quantify the influence of decision-maker's psychological behaviors on decision-making results. Third, COVID-19 is taken as an example for empirical analysis to verify the feasibility of the emergency response mechanism. Prospect theory is used to calculate the combined cumulative prospect value of each alternative. Ultimately, the best contingency plan can be selected. The research results are of great significance for decision-makers to quickly collect knowledge and respond to similar emergencies and provide a scientific basis for preventing and controlling public health emergencies.

## Emergency decision mechanism for public health emergency

### Dynamic scenario deduction for public health emergency

**Figure 1:**
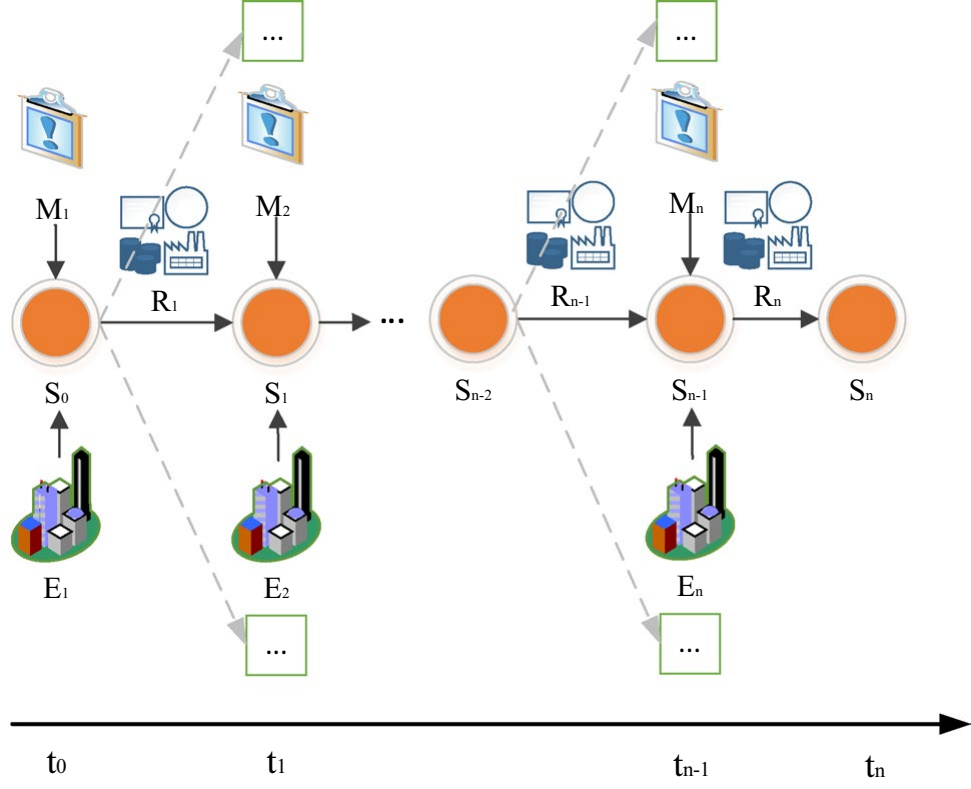
The evolution process of the emergency scenario.

Constructing a major public emergency scenario shall analyze the evolution law, sort out the emergency tasks, evaluate the emergency capability of the major emergencies that may occur and the expected risks, and improve the scheme and strengthen the emergency preparedness accordingly to enhance the emergency capability. Usually, the evolution of an emergency scenario is associated with constituent elements of the scenario and the relationship among these elements, which is a dynamic process^12^. The elements include scenario state (S), the external environment (E), emergency measures (M), and emergency resources (R). The emergency deduction process is: in the current S, the scenario state will change under the influence of E, the constraints of R, and the intervention of M, switching from S to S_1_ to complete a complete scenario evolution process. The time when each scenario state occurs is defined as $t_{0} ,t_{1} ,t_{2} ...t_{n}$ to construct the corresponding emergency scenario evolution process, as shown in Fig. 1. Due to changes in the external environment and the adjustment of emergency resource constraints, the emergency measures taken at different stages are different, making the emergency scenario evolution unknown and unpredictable^13–15^. Hence, compared with the current scenario state, the next scenario state has a variety of possibilities until the last time *t*_*n*_ at which the emergency scenario disappears. At this time, the evolution process ends, indicating that the entire emergency process is over.

According to the above analysis, the scenario evolution of emergencies is a dynamic and changeable continuous process, so that the corresponding emergency decision should also be based on the current scenario state. The emergency scenario can be changed into the next state as soon as possible by taking appropriate measures. Generally, to ensure the rationality of the decision, decision-makers shall evaluate the effect of the previous emergency before deciding to determine whether the emergency goal is reached. If the emergency is not controlled or even deteriorates in the last emergency process, the next decision will be made to adjust the scheme in a targeted manner so that the emergency evolves toward an optimistic development path.

### A-DRE based on rough set attribute reduction

Public health emergency has key characteristics such as instantaneity, uncertainty, and lack of information. Its data are often complex and changeable, bringing serious obstacles to data acquisition and analysis^16^. Only through attribute reduction on public health emergency data and supplementing missing data can the data features be extracted accurately^17^. In turn, data can be processed in a targeted manner, helping decision-makers formulate emergency schemes quickly efficiently. The rough set theory in mathematical tools is chosen in the present work to solve the uncertainty problem. The advantage of the rough set is that, besides the dataset, the uncertainty of the problem can be described and dealt with objectively without any prior knowledge.

Suppose that *U* is a non-empty finite set of objects of interest, that is, the domain of discourse. Any subset of the domain *U* is called abstract knowledge about *U*, and each concept in *U* represents an information particle. The rough set theory discusses the knowledge that can be divided or covered on *U*. While solving actual problems, what is dealt with is not a single division on *U* but a family of divisions to form a knowledge base $K \in \left( {U,R} \right)$. Two subsets can be defined for each subset *X* and an equivalent relation *R*, representing the lower and upper approximation sets of *X*, respectively:

1 $$\underline{R} X = \cup \left\{ {Y \in U/R|Y \subseteq X} \right\}$$

2 $$\overline{R} X = \cup \left\{ {Y \in U/R|Y \cap X \ne \emptyset } \right\}$$

Suppose that a cover on *U* is *C*; in that case, the corresponding minimum and maximum descriptions and neighborhoods can be defined as:

3 $$Md\left( x \right) = \left\{ {K \in C|x \in K \wedge \left( {\forall S \in C \wedge x \in S \wedge S \subseteq K} \right) \Rightarrow K = S} \right\}$$

4 $$Mx\left( x \right) = \left\{ {K \in C|x \in K \wedge \left( {\forall S \in C \wedge x \in S \wedge S \subseteq S} \right) \Rightarrow K = S} \right\}$$

5 $$N\left( x \right) = \cap \left\{ {K \in C|x \in K} \right\}$$

The essence of attribute reduction on a knowledge base is to ensure the unchanged classification ability of the decision table. It deletes the non-principal knowledge and ultimately retains the key condition attributes. The original rough set theory is based on the equivalence relation model, restricting the range of data processing. In this case, the coverage rough set theory is proposed, and the approach to process the relationships among attributes is shifted to the coverage mode. Hence, the attribute reduction problem can be analyzed based on the coverage rough set. Suppose that $\Delta = \left\{ {C{}_{1},C{}_{2}...C{}_{n}} \right\}$ represents the family coverage on *U*, and the coverage decision table is $S = \left( {U,\Delta ,D} \right)$. In that case, the ability of a coverage *C*_*i*_ to correctly classify the objects in *U* based on the decision attribute *D* is the positive domain of *S*, which can be described as:

6 $$pos_{{C{}_{i}}} D = \mathop \cup \limits_{X \in U/D} \underline{R} X$$

Through association rule mining, the internal connection between data can be discovered effectively, and the knowledge that is conducive to correct decision-making can be output. However, traditional association rule mining cannot handle datasets with missing information and redundant data regarding emergencies. In contrast, the rough set theory is more targeted for emergency knowledge acquisition^18–20^. Rules represent the connection between large amounts of data. The higher the support of the decision rules, the more samples are covered, indicating the higher the reliability of the decision rules. The coverage of a decision rule refers to the proportion of the object in the corresponding decision category. The higher the coverage, the greater the influence of the rule’s antecedents on the subsequent. A set of ordered rules is also called a decision table. The test record is classified by the rule covering the highest rank of the record, avoiding the class conflict caused by the prediction of multiple classification rules.

A coverage decision table is expressed as $\left( {U,\Delta ,D} \right)$, $U = \left\{ {x{}_{1},x{}_{2}...x{}_{n}} \right\}$, $S = \left( {U,\Delta ,D} \right) = \left\{ {C_{k} \in \Delta |\exists X_{i} \in U/Ds.t.} \right\}$ is the effective approximation set off $\left( {U,\Delta ,D} \right)$, where $C_{k}$ is the effective approximation element. There are the following relationships for any $x{}_{i} \in \cup S\left( {U,\Delta ,D} \right)$:

7 $$r\left( {x{}_{i}} \right) = \left\{ {C \in \Delta |\exists C_{k} \in S\left( {U,\Delta ,D} \right)s.t. \, x{}_{i} \in C_{k} \in C} \right\}$$

8 $$R\left( {U,\Delta ,D} \right) = \left\{ {r\left( {x{}_{i}} \right)|x{}_{i} \in \Delta } \right\}$$

In (7) and (8), $r\left( {x{}_{i}} \right)$ refers to the set of $x{}_{i}$, and $R\left( {U,\Delta ,D} \right)$ is the major family of the decision table $\left( {U,\Delta ,D} \right)$.

The attribute reduction of the decision table can be achieved through the family method based on the approximate space coverage rough set model. The specific reduction process will be explained through the following example. If a decision table $S = \left( {U,A,V,f} \right)$ is known, the corresponding attribute representation will be symbolic data, where $U = \left\{ {x{}_{1},x{}_{2},x{}_{3},x{}_{4},x{}_{5},x_{6} } \right\}$ is a set of objects, $A = \left\{ {a{}_{1},a{}_{2},a{}_{3},a{}_{4},a{}_{5}} \right\}$ is a set of objects, and $D = \left\{ d \right\}$ is a decision attribute. According to the method of information granule generation, the decision table is processed, and each attribute can be formed into a corresponding coverage, which can be expressed as:

9 $$C_{1} = \left\{ {\left\{ {x{}_{1},x{}_{2}} \right\},\left\{ {x{}_{3},x{}_{4}} \right\},\left\{ {x{}_{5},x{}_{6}} \right\}} \right\}$$

10 $$C_{2} = \left\{ {\left\{ {x{}_{1},x{}_{2},x{}_{4}} \right\},\left\{ {x{}_{3}} \right\},\left\{ {x{}_{1},x{}_{2},x{}_{4},x{}_{5}} \right\},\left\{ {x{}_{4},x{}_{5}} \right\},\left\{ {x{}_{6}} \right\}} \right\}$$

11 $$C_{3} = \left\{ {\left\{ {x{}_{1}} \right\},\left\{ {x{}_{2},x{}_{3},x{}_{4}} \right\},\left\{ {x{}_{5},x{}_{6}} \right\}} \right\}$$

12 $$C_{4} = \left\{ {\left\{ {x{}_{1},x{}_{2},x{}_{4}} \right\},\left\{ {x{}_{1},x{}_{2},x{}_{3},x{}_{4}} \right\},\left\{ {x{}_{3},x{}_{4},x{}_{5},x{}_{6}} \right\},\left\{ {x{}_{3},x{}_{5},x{}_{6}} \right\}} \right\}$$

13 $$C_{5} = \left\{ {\left\{ {x{}_{1},x{}_{2}} \right\},\left\{ {x{}_{1},x{}_{3}} \right\},\left\{ {x{}_{1},x{}_{2},x{}_{3}} \right\},\left\{ {x{}_{4}} \right\},\left\{ {x{}_{5},x{}_{6}} \right\}} \right\}$$

14 $$U/D = \left\{ {\left\{ {x{}_{1},x{}_{2},x{}_{3}} \right\},\left\{ {x{}_{4},x{}_{5},x{}_{6}} \right\}} \right\}$$

The set that effectively approximates each object $x{}_{i}$ in the set $S = \left( {U,\Delta ,D} \right)$ is:

15 $$r\left( {x{}_{1}} \right) = \left\{ {C{}_{1},C{}_{3},C{}_{5}} \right\}$$

16 $$r\left( {x{}_{2}} \right) = \left\{ {C{}_{1},C{}_{5}} \right\}$$

17 $$r\left( {x{}_{3}} \right) = \left\{ {C{}_{2},C{}_{5}} \right\}$$

18 $$r\left( {x{}_{4}} \right) = \left\{ {C{}_{2},C{}_{5}} \right\}$$

19 $$r\left( {x{}_{5}} \right) = \left\{ {C{}_{1},C{}_{2},C{}_{3},C{}_{5}} \right\}$$

20 $$r\left( {x{}_{6}} \right) = \left\{ {C{}_{1},C{}_{2},C{}_{3},C{}_{5}} \right\}$$

The family of decision table $S = \left( {U,A,V,f} \right)$ can be expressed as:

21 $$R\left( {U,\Delta ,D} \right) = \left\{ {\left\{ {C{}_{1},C{}_{3},C{}_{5}} \right\},\left\{ {C{}_{1},C{}_{5}} \right\},\left\{ {C{}_{2},C{}_{5}} \right\},\left\{ {C{}_{1},C{}_{2},C{}_{3},C{}_{5}} \right\}} \right\}$$

The function corresponding to the coverage decision table $S = \left( {U,A,V,f} \right)$ can be expressed as:

22 $$\begin{aligned} f\left( {U,\Delta ,D} \right) = & \left( {C{}_{1} \vee C{}_{3} \vee C{}_{5}} \right) \wedge \left( {C{}_{1} \vee C{}_{5}} \right) \wedge \left( {C{}_{2} \vee C{}_{5}} \right) \wedge \\ & \left( {C{}_{1} \vee C{}_{2} \vee C{}_{3} \vee C{}_{5}} \right) = \left( {C{}_{1} \wedge C{}_{2}} \right) \vee C{}_{5} \\ \end{aligned}$$

At this time, the attribute reduction form of decision $S = \left( {U,A,V,f} \right)$ is:

23 $$red\left( \Delta \right) = \left\{ {\left\{ {C{}_{1},C{}_{2}} \right\},C{}_{5}} \right\}$$

**Figure 2:**
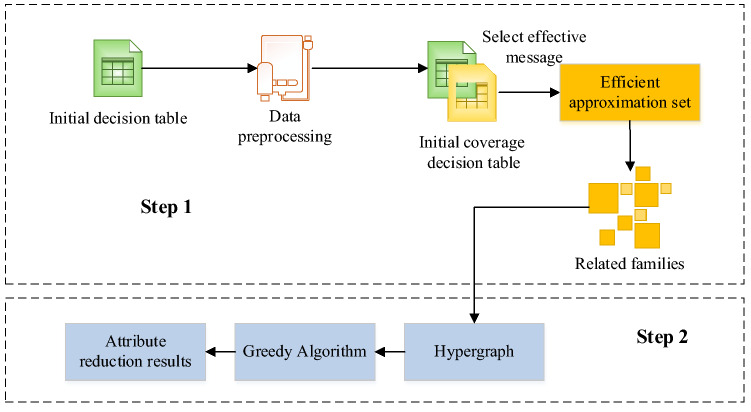
Basic procedures of A-DRE.

A Decision Rule Extraction Algorithm (A-DRE) considering attribute cost is proposed based on the family method in the present work. According to the family theory, this algorithm first obtains the family of the decision table and then transforms it into a hypergraph. Next, it uses the greedy algorithm to obtain the minimum vertex cover, and finally, obtains the attribute reduction result of the decision table. While applying the greedy algorithm, a mathematical model is first established to describe the problem, and the problem to be solved is divided into several sub-problems. Then, each sub-problem is solved to obtain the optimal local solution of the sub-problem. Eventually, the optimal local solution of the sub-problem is combined into a solution to the original problem. The basic framework of A-DRE is demonstrated in Fig. 2.

### Multi-attribute emergency decision model based on prospect theory

From a psychological perspective, when making decisions under uncertain conditions, decision-makers may preset a reference standard in their minds^21^. The connotation of prospect theory is the gap between the decision result and the expected result, not just the result itself. The mathematical function when people measure the gains and losses of decision-making is expressed as:

24 $$U = w\left( {p_{1} } \right)v\left( {x_{1} } \right) + w\left( {p_{2} } \right)v\left( {x_{2} } \right) + ...w\left( {p_{n} } \right)v\left( {x_{n} } \right)$$

**Figure 3:**
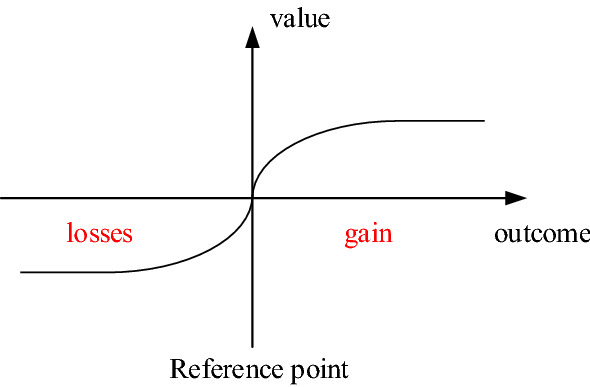
Trend of the value function.

In (24), $x_{i}$ represents all possible results, $p_{i}$ refers to the probability of these results, *v*, as a value function, represents the relative value of different results in the minds of decision-makers. Figure 3 illustrates the trend of the value function. The asymmetry of the value function suggests that the absolute value of a loss result is greater than the absolute value of the profit result.

An example is the specific decision-making process, including three stages of editing, evaluation, and selection^22–24^. Editing is a process in which decision-makers set the criteria for gains and losses. If the judgment result is better than the reference point, the result will be defined as profit. Evaluation is the process of calculating the value of the prospect using the value function, which reflects the subjective value of the result. In the final selection process, the decision-maker compares the prospect values of all schemes and takes the scheme with the largest prospect value as the final decision result. The decision-making process embodies the characteristics of decision-makers risk appetite and gain–loss sensitivity in an uncertain environment. The value function $v\left( {\Delta x_{i} } \right)$ and the probability weight function $\pi \left( {p_{i} } \right)$ jointly determine the prospect value *V*. The model can be expressed as:

25 $$V = \sum\limits_{i = 1}^{k} {\pi \left( {p_{i} } \right)v\left( {\Delta x_{i} } \right)}$$

26 $$v\left( {\Delta x_{i} } \right) = \left\{ \begin{gathered} v\left( {\Delta x_{i} } \right)^{\alpha } , \quad \Delta x_{i} \ge 0 \hfill \\ - \lambda \left( { - \Delta x_{i} } \right)^{\beta } {,}\quad \Delta x_{i} < 0 \, \hfill \\ \end{gathered} \right.$$

27 $$\pi^{ + } \left( p \right) = \frac{{p^{\chi } }}{{\left[ {p^{\chi } + \left( {1 - p} \right)^{\chi } } \right]^{1/\chi } }}$$

28 $$\pi^{ - } \left( p \right) = \frac{{p^{\delta } }}{{\left[ {p^{\delta } + \left( {1 - p} \right)^{\delta } } \right]^{1/\delta } }}$$

In (25)–(28), $v$ represents the value function, $\pi$ represents the probability weight function, $\alpha ,\beta$ assess the degree of the risk appetite of decision-makers, $\lambda$ indicates the degree of decision-makers aversion to loss, $\pi^{ + } \left( p \right)$ and $\pi^{ - } \left( p \right)$ represent the probability weights of gain and loss, $\chi$ and $\delta$ are the risk level at the time of gain and the risk attitude at the time of loss.

A processing scheme is given for the specific execution process after decision-making regarding each state of a public health emergency. These processes can be descriptive qualitative information or formal quantitative analysis information. The decision-making model needs to process the scheme information in intervals to form the gray data subordination range value based on the attribute state as the amount of data during decision-making. In this way, the final decision result becomes quantified and rational. The gray emergency decision matrix of each rule property is shown in Table 1.

**Table 1:** Gray emergency decision matrix of each rule attribute.

Rule property	Status	Probability	Probability			
			$A_{1}$	$A_{2}$	…	$A_{n}$
$U_{1}$	$$\begin{gathered} S_{1} \hfill \\ S_{2} \hfill \\ ... \hfill \\ S_{{l_{1} }} \hfill \\ \end{gathered}$$	$$\begin{gathered} p_{1}^{1} \hfill \\ p_{1}^{2} \hfill \\ ... \hfill \\ p_{1}^{{l_{1} }} \hfill \\ \end{gathered}$$	$$\begin{gathered} x_{11}^{1} \left( \otimes \right) \hfill \\ x_{21}^{1} \left( \otimes \right) \hfill \\ ... \hfill \\ x_{{l_{1} 1}}^{1} \left( \otimes \right) \hfill \\ \end{gathered}$$	$$\begin{gathered} x_{12}^{1} \left( \otimes \right) \hfill \\ x_{22}^{1} \left( \otimes \right) \hfill \\ ... \hfill \\ x_{{l_{1} 2}}^{1} \left( \otimes \right) \hfill \\ \end{gathered}$$	…	$$\begin{gathered} x_{1n}^{1} \left( \otimes \right) \hfill \\ x_{2n}^{1} \left( \otimes \right) \hfill \\ ... \hfill \\ x_{{l_{1} n}}^{1} \left( \otimes \right) \hfill \\ \end{gathered}$$
$U_{2}$	$$\begin{gathered} S_{1} \hfill \\ S_{2} \hfill \\ ... \hfill \\ S_{{l_{2} }} \hfill \\ \end{gathered}$$	$$\begin{gathered} p_{2}^{1} \hfill \\ p_{2}^{2} \hfill \\ ... \hfill \\ p_{2}^{{l_{2} }} \hfill \\ \end{gathered}$$	$$\begin{gathered} x_{11}^{2} \left( \otimes \right) \hfill \\ x_{21}^{2} \left( \otimes \right) \hfill \\ ... \hfill \\ x_{{l_{2} 1}}^{2} \left( \otimes \right) \hfill \\ \end{gathered}$$	$$\begin{gathered} x_{22}^{2} \left( \otimes \right) \hfill \\ x_{22}^{2} \left( \otimes \right) \hfill \\ ... \hfill \\ x_{{l_{2} 2}}^{2} \left( \otimes \right) \hfill \\ \end{gathered}$$	…	$$\begin{gathered} x_{1n}^{2} \left( \otimes \right) \hfill \\ x_{2n}^{2} \left( \otimes \right) \hfill \\ ... \hfill \\ x_{{l_{2} n}}^{2} \left( \otimes \right) \hfill \\ \end{gathered}$$
…	…	…	…	…	…	…
$U_{n}$	$$\begin{gathered} S_{1} \hfill \\ S_{2} \hfill \\ ... \hfill \\ S_{{l_{n} }} \hfill \\ \end{gathered}$$	$$\begin{gathered} p_{n}^{1} \hfill \\ p_{n}^{2} \hfill \\ ... \hfill \\ p_{n}^{{l_{n} }} \hfill \\ \end{gathered}$$	$$\begin{gathered} x_{11}^{n} \left( \otimes \right) \hfill \\ x_{21}^{n} \left( \otimes \right) \hfill \\ ... \hfill \\ x_{{l_{n} 1}}^{n} \left( \otimes \right) \hfill \\ \end{gathered}$$	$$\begin{gathered} x_{1n}^{n} \left( \otimes \right) \hfill \\ x_{2n}^{n} \left( \otimes \right) \hfill \\ ... \hfill \\ x_{{l_{n} n}}^{n} \left( \otimes \right) \hfill \\ \end{gathered}$$	…	$$\begin{gathered} x_{1m}^{n} \left( \otimes \right) \hfill \\ x_{2m}^{n} \left( \otimes \right) \hfill \\ ... \hfill \\ x_{{l_{n} m}}^{n} \left( \otimes \right) \hfill \\ \end{gathered}$$

Under an actual public health emergency, the timelier the emergency response is, the better it can prevent the loss from spreading^25^. In the face of major public health emergencies, the probability of an emergency state is usually raised by a group of decision-makers^26,27^. The subjective probability weight of the decision-maker is obtained based on the probability weight function, which represents the psychological perception of each event state to the decision-maker. Gathering the information on the comprehensive prospect value matrix of different decision-makers is a vital link to attain the comprehensive opinions of the group. Ideally, if the individual matrix information is completely consistent with the group’s collective information, the decision-making group has completely accepted the opinions of the decision-maker. The equation to calculate the similarity between the group consensus matrix $V^{*}$ and the *k*-th decision-maker matrix $V_{ij}^{k}$ is as follows:

29 $$d_{k} \left( {V^{k} ,V^{*} } \right) = \sum\limits_{i = 1}^{m} {\sum\limits_{j = 1}^{n} {d\left( {V_{ij}^{k} ,V_{ij}^{*} } \right)} }$$

The group decision information integrates the wisdom of the decision-makers; however, since the determination of each scheme is based on different criteria, it is necessary to weigh the comprehensive prospect value of the final decision scheme. The optimal set matrix $$V^{**} = \left( {V_{ij}^{**} } \right)_{m \times n}$$ of the group can be obtained by weighting the comprehensive prospect value of each decision-maker’s decision scheme, and finally, the comprehensive prospect value matrix is:

30 $$V^{**} = \left( {V_{ij}^{**} } \right)_{m \times n} = \left( \begin{gathered} \left[ {V_{11}^{**L} ,V_{11}^{**U} } \right]\left[ {V_{12}^{**L} ,V_{12}^{**U} } \right]...\left[ {V_{1n}^{**L} ,V_{1n}^{**U} } \right] \hfill \\ \left[ {V_{21}^{**L} ,V_{21}^{**U} } \right]\left[ {V_{22}^{**L} ,V_{22}^{**U} } \right]...\left[ {V_{2n}^{**L} ,V_{2n}^{**U} } \right] \hfill \\ ... \hfill \\ \left[ {V_{m1}^{**L} ,V_{m1}^{**U} } \right]\left[ {V_{m2}^{**L} ,V_{m2}^{**U} } \right]...\left[ {V_{mn}^{**L} ,V_{mn}^{**U} } \right] \hfill \\ \end{gathered} \right)$$

### Framework of multi-department emergency decision

**Figure 4:**
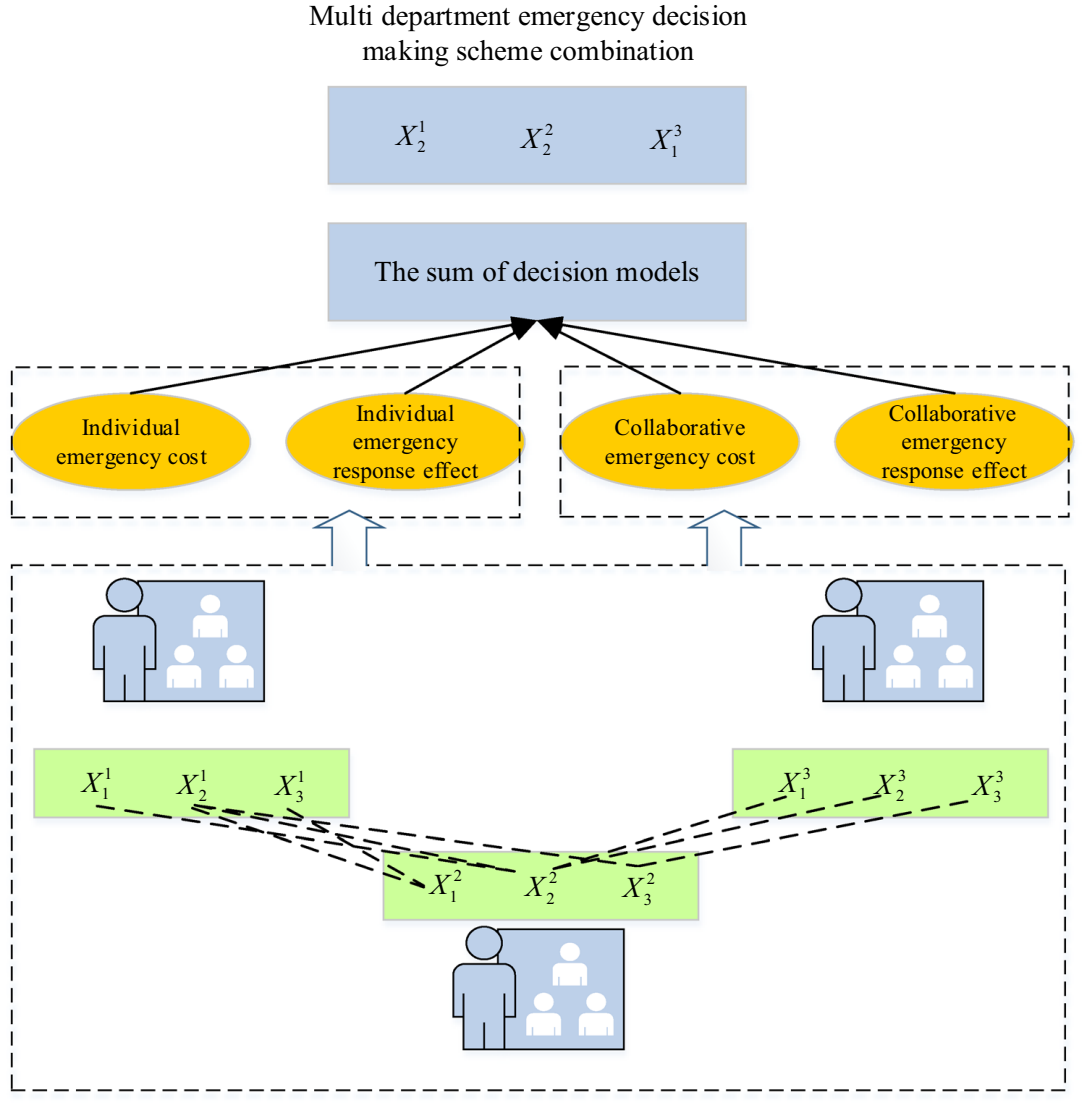
Selection process of multi-department emergency decision.

The feature of the emergency decision is adjusting the emergency scheme in time according to the changes in the environment. It is non-procedural decision-making, whose process must highlight timeliness^28^. The public health emergency requires multiple departments to participate in the emergency process and gather expert knowledge in multiple fields. After the multi-department decision-making gets summarized, the overall optimization is performed for effective group decision-making. Whether the selection of emergency decision indicators is appropriate directly affects the evaluation of the final decision scheme^29,30^. Hence, the selection of indicators should grasp the principles of systematicness, measurability, comparability, and effectiveness. Combining the emergency decision scheme between multi-department should consider not only the individual emergency cost and emergency effect but also the collaborative cost and synergistic effect of the multi-department emergency decision scheme. The complementarity of resources between departments, the convenience of communication, and the degree of personnel collaboration will affect the final emergency response during the coordinated decision-making between different departments. The selection process of the multi-department emergency decision is described as the structure in Fig. 4. In Fig. 4, $X_{i}^{k}$ represents the *i*-th emergency decision scheme of department *k*.

### Algorithm analysis and application instance

Five public datasets in the University of California Irvine (UCI) database are selected as test data to validate the performance of the proposed A-DRE based on the rough set theory, namely wbpc, wdbc, segment, inequality, and texture. Two methods based on Neighborhood Rough Set (NRS) and Information Entropy (HANDI) are selected for comparison, and both Support Vector Machine (SVM) and Decision Tree (ID3) classifiers are employed to measure the classification accuracy of the data. The operating system of the equipment is Windows 7, the processor is Intel Core i5, and the memory is 8 GB. The experiment and data analysis is completed on MATLAB.

Since the first case was discovered in Wuhan, China, in December 2019, COVID-19 has developed rapidly, making it the most difficult public health emergency since the founding of the People’s Republic of China. Based on the public data, COVID-19 has a high incidence, persistence, and high infectiousness. If an emergency decision can be made in a timely and effective manner for emergency response, the harm caused by the pandemic can be reduced as much as possible, and the epidemic can be prevented from further expanding. The COVID-19 Dataset^31^ records the disease data of 21 provinces and cities across China from January to April 2020, a total of 81 event information, including 15 numerical condition attributes. The decision attribute is a specific “emergency response level,” which is divided into three levels. Because some conditional attribute information in the original data is missing, only conditional attributes that do not affect attribute reduction and rule extraction are retained.

In model decision-making, it is necessary to clarify the influencing factors that can be used for quantitative analysis of the model and ensure the retention and accuracy of the information during quantification. Among the quantifiable influencing factors, explicit factors such as the number of equipment that can be used in emergency rescue, the number of medical staff, the number of infected people, and the area range are important characteristic data that need to be determined at any time. According to the emergency decision modeling idea, based on these data, the associated dataset structure and state association can be represented. The graded evaluation information construction approach can obtain the corresponding matrix construction mode so that these data can be calculated and accurately used in the specific decision-making process to formulate an emergency rescue scheme or form an emergency rescue system.

## Results and discussion

### A-DRE complexity and effectiveness analysis

**Figure 5:**
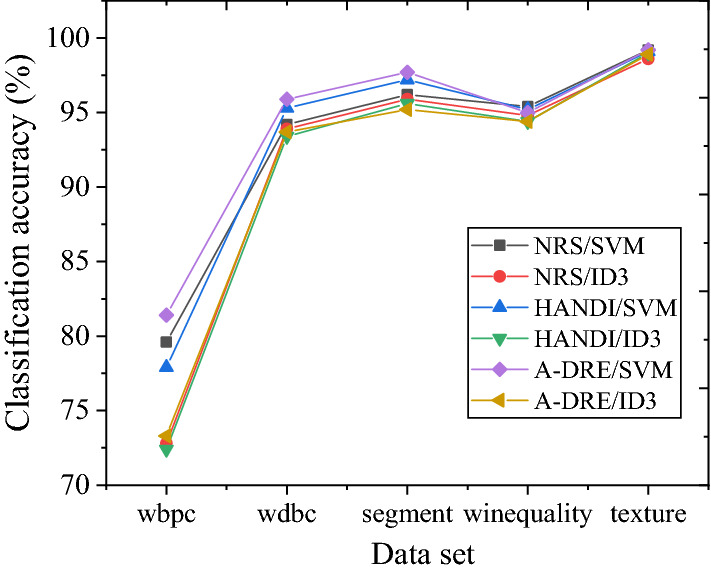
Classification accuracy of different data reduction algorithms.

**Figure 6:**
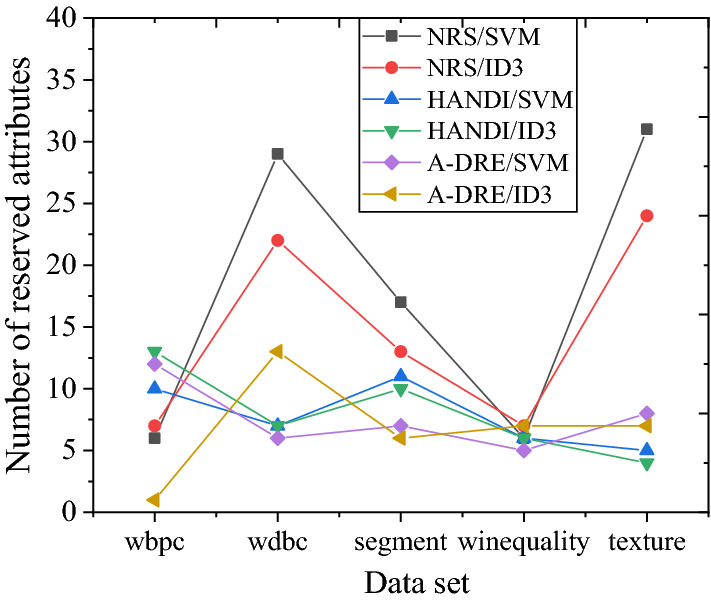
Number of attributes retained by different data reduction algorithms.

In the course of this experiment, all algorithms can calculate the reduction results of five datasets. The classification accuracy results of several algorithms for different datasets are presented in Fig. 5. The accuracy can be maintained or improved compared to the initial classification accuracy using A-DRE to reduce the dataset. Compared with the other two algorithms, the final classification accuracy difference is small, proving the algorithm’s effectiveness. Figure 6 displays the number of attributes retained by different algorithms after attribute reduction on the dataset. The comparison of several data reduction algorithms reduces the scale of conditional attributes in the original dataset to varying degrees. In comparison, the average number of key attributes extracted by A-DRE is less. Hence, A-DRE can efficiently filter out important attributes from the original dataset, and the complexity of the algorithm is low. Therefore, applying A-DRE to the attribute reduction of large-scale data in public health emergencies can efficiently and accurately extract the key factors of the data, thereby determining the hazard level of emergencies and the development of rescue activities.

### Attribute reduction for COVID-19 emergency response dataset

**Figure 7:**
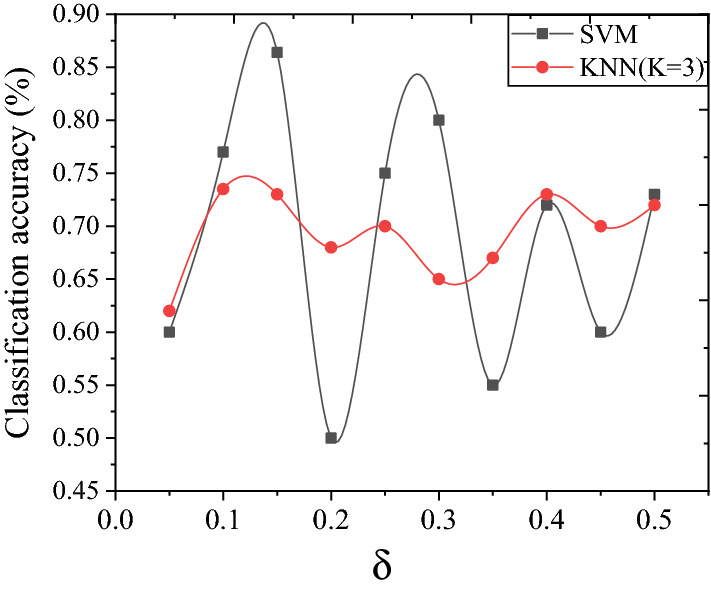
A-DRE attribute reduction results in the COVID-19 Dataset.

After the original dataset of COVID-19 emergency response is preprocessed, A-DRE is utilized for attribute reduction, and the reduction result is summarized in Fig. 7. When $\delta { = }0.10$ the classification accuracy under the KNN classifier reaches 73.5%; when $\delta { = }0.15$ the classification accuracy under the SVM classifier reaches 86.4%. The classification accuracy based on the KNN classifier is more stable than SVM. The above reduction results prove that A-DRE can perform attribute reduction on the COVID-19 emergency response dataset and effectively reduce the sum of the attribute costs of collecting information.

Figure 7 proves that the A-DRE algorithm can not only perform attribute reduction on the COVID-19 emergency response data set but also ensure that the sum of the cost of the reduced attribute set is small. The attribute-reduced dataset makes the relationship between condition attributes and emergency response levels more straightforward and can effectively reduce the sum of attribute costs for collecting relevant information.

### COVID-19 emergency decision scheme based on multi-department collaboration

In the face of the outbreak and spread of COVID-19, when multi-department coordinated decision-making is required, the departments involved include emergency command centers, emergency service agencies, and emergency measures implementation departments. First, a collaborative network and a collaborative matrix between departments should be established. By analyzing the severity, controllability, and scope of COVID-19, the initial weight vector of the evaluation criteria, such as emergency decision cost and effect, can be determined.

Suppose that a COVID-19 command center responds to the pandemic based on five indicators; a case of COVID-19 occurs in a community on a day. In that case, the decision center needs to choose from three alternative schemes as the emergency decision: x_1_, dispatching a medical team and an emergency ambulance; x_2_, based on x_1_, sending an additional medical expert and an emergency medical equipment; x_3_, based on x_2_, sending an additional medical emergency protection team. Three indicators of the alternative schemes are evaluated to determine the optimal rescue scheme: c_1_ health status of the infected case, c_2_ the number of rescuers, c_3_ costs of rescue equipment and labor. Besides the cost indicator c_3_, the rest are gain indicators. If the emergency assistance for COVID-19 is divided into 4-time frames $t_{1} \sim t_{4}$, the attribute weight of the indicator is $\omega = \left( {0.4,0.45,0.15} \right)$, and the expected vector of attributes is $R = \left( {6,\left[ {5,11} \right],\left[ {G,M} \right]} \right)$. The initial evaluation information is summarized in Table 2.

**Table 2:** Initial evaluation information.

Time	Alternative scheme	c_1_	c_2_	c_3_
t_1_	x_1_	4	[2,5]	[VG,MG]
	x_2_	4	[4,7]	[G,MG]
	x_3_	5	[3,6]	[MG,MP]
t_2_	x_1_	3	[4,6]	[G,MG]
	x_2_	6	[6,12]	[MG,M]
	x_3_	5	[5,9]	[MG,M]
t_2_	x_1_	3	[4,6]	[G,MG]
	x_2_	5	[6,11]	[MG,M]
	x_3_	6	[8,15]	[MG,M]
t_3_	x_1_	3	[5,6]	[G,MG]
	x_2_	7	[9,12]	[M,MP]
	x_3_	6	[7,10]	[MG,M]

Through calculations, the final cumulative prospect values are $U\left( {x_{1} } \right) = - 1.3367$, $U\left( {x_{2} } \right) = 0.0091$, and $U\left( {x_{3} } \right) = - 0.0901$. The final cumulative prospect values $x_{1}$ and $x_{3}$ are negative, suggesting this emergency decision scheme is at a loss for the decision-maker relative to the reference point. In particular, $x_{1}$ has fewer emergency reserve resources and weak emergency response capability, and the timeliness of emergency response cannot be guaranteed; in contrast, $x_{3}$ has sufficient emergency resources and strong emergency response capabilities; however, it significantly increases emergency costs while ensuring the timeliness of emergency response. As the optimal choice $x_{2}$ can save part of the emergency cost while meeting the timeliness requirements of emergency decisions.

### Emergency management of public health emergencies supported by blockchain technology

The essence of the blockchain is to achieve data trust between unrelated parties through encryption and consensus algorithms to ensure the consistency, non-tampering, and traceability of shared data. This can break information islands, accelerate the credible sharing of data, and help existing technologies to build a more in-depth, collaborative, and shared emergency management information system across departments and regions. As a result, it enables the data to be realized from perception to cognition and provides an accurate basis for emergency management decision-making. Meanwhile, all elements involved in emergency management are virtualized under the trusted data collaboration system constructed by the blockchain. It can fully simulate and predict all possible situations and respond to them. Finally, it will use a large amount of real-time data to optimize decision-making for continuous learning and correction.

Emergency management involves a wide range of departments. It is highly complex and has a high degree of difficulty. Overall coordination has become a key problem for emergency command. In terms of material allocation, at the beginning of the outbreak of the COVID-19 epidemic, the material allocation in the key epidemic prevention and control areas was disordered, and there was a shortage of epidemic prevention materials such as medical masks, protective clothing, and thermometers to varying degrees. Blockchain technology can realize the transparency and check of information in the whole process of emergency material transportation. In response to traffic control caused by the epidemic, it can check the exact location and status of materials at any time, and make route changes in time. When there is an emergency that supplies are occupied, it can also quickly determine the responsible party and save the evidence. In addition, the existing system has the insufficient predictive ability for public health emergencies, and cannot carry out pre-warning and rapid improvement of measures. Emergency management decision-making must become an agile response learning organization. Combining human and machine learning can improve the scientificity and predictive accuracy of emergency management decision-making.

## Conclusion

The rationality of emergency measures and emergency targets can determine the development direction of public health emergencies. Based on its nature, information on public health emergencies also presents characteristics such as complexity, dynamics, and missing values. Therefore, a dynamic decision model based on rough set attribute reduction and prospect theory is established regarding the dynamic multi-attribute risky public health emergency decisions, and an A-DRE considering attribute cost is proposed. The different risky psychology and gain and loss sensitivity of decision-makers can affect the final decision result. Prospect theory is employed to calculate each alternative scheme’s comprehensive cumulative prospect value to select the best emergency solution. Based on the rough set theory and the attribute cost, the knowledge acquisition of emergencies can extract the decision rules with better classification ability and lower cost in the decision table. Eventually, the actual application of A-DRE is validated through the case analysis of the COVID-19 dataset. Because formulating an emergency decision scheme requires coordination between different departments, their coordination relationships and the compatibility of decision schemes are also analyzed to ensure an effective final emergency decision. Appropriate emergency measures and emergency goals can delay the deterioration of disease evolution, buy time for emergency response, and reduce the personnel and economic losses caused by the disease. The research on COVID-19 is of practical value in dealing with the current situation where the disease is not fully controlled. The downside is that the dynamic nature of public health emergencies does not consider the significant impact on decision-making. Therefore, in the following work, the feedback after implementing emergency decision-making will be incorporated into the evaluation criteria to adjust the emergency response plan promptly.
